# A case of phantom pain and stump pain that was effectively controlled by ultrasound‐guided ulnar and median peripheral nerve blocks

**DOI:** 10.1002/ccr3.7672

**Published:** 2023-07-10

**Authors:** Shunya Sekiguchi, Yusuke Ishida, Yosuke Fujita, Mikiko Tomino, Kiyoshige Ohseto

**Affiliations:** ^1^ Department of Anesthesiology Tokyo Medical University Tokyo Japan

**Keywords:** allodynia, median nerve block, phantom pain, stump pain, ulnar nerve block

## Abstract

Phantom limb pain and stump pain are often intractable, and their incidences are relatively high. We report a case of a patient with phantom limb and stump pain of the finger, who was successfully treated by peripheral nerve blocks. The patient was a male truck driver in his fifties, who had his left annular finger amputated in an accident 2 years previously. Owing to poor pain control at the stump of his finger, he was referred to our department. The initial examination revealed pain about numerical rating scale (NRS) 6/10 in the left annular finger transection as well as allodynia. Although some pain relief had been observed with postoperative medication, he still had persistent resting pain of about NRS 4/10. Therefore, blocks of the ulnar nerve and median nerve were performed. After the blocks were performed, the pain improved to NRS 1 to 2/10, and pain upon movement also almost disappeared. Peripheral nerve blocks can be a useful treatment modality for phantom limb pain and stump pain in the fingers, as in this case.

## Graphical abstract

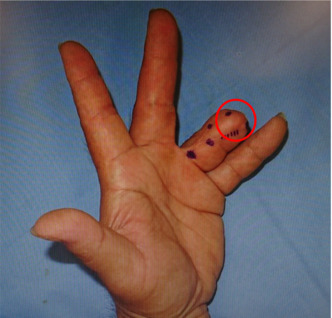

## 1 BACKGROUND

Phantom limb pain and stump pain are commonly observed in patients after limb amputation, and these pains are difficult to control and are often refractory.^1^ Here, we report a case of a patient with chronic phantom limb pain and stump pain, in whom peripheral nerve blocks were effective. We obtained written informed consent from the patient to publish this case report.

## 2 CASE PRESENTATION

**FIGURE 1:**
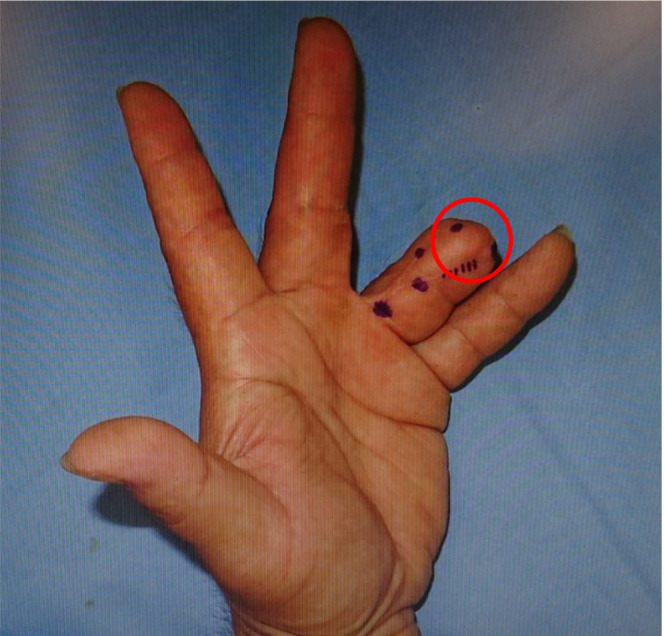
Condition of the patient's finger after amputation. The patient continued to have pain at the left annular transection after surgery. His pain was localized to the left proximal phalanx, and allodynia was also present over the phalanx (red circle). He had numbness at the site of pain, and also recognized a decrease in sensation.

The patient was a male truck driver in his fifties, who had his left annular finger amputated in an accident 2 years previously, and since that time, he had been experiencing pain at the distal end of the amputated finger. The numerical rating scale (NRS) was 6/10 at rest and 8/10 with movement. (NRS requires the patient to rate their pain on a defined scale. 0 is no pain and 10 is the worst pain imaginable.) Seven months after the accident, the patient underwent VY flap surgery at his previous clinic. He was referred to the Department of Plastic Surgery at our hospital because of stump neuroma 4 months previously, and neuroma resection was performed (Figure 1). Because the patient continued to have pain at the left annular transection after surgery, tramadol hydrochloride/acetaminophen combination tablets were prescribed. However, the patient was unable to take this orally at his own discretion owing to concerns about drowsiness and other adverse drug reactions while driving. Owing to poor pain control at the stump of his finger, he was referred to our department. At the time of the initial examination, his pain was localized to the left proximal phalanx, and allodynia was also present over the phalanx. (Allodynia is pain due to a stimulus that does not normally provoke pain.) The NRS was 4/10 at rest and 6/10 with movement, with a tendency for the pain to worsen. He had numbness at the site of pain, and also recognized a decrease in sensation. His Self‐Rating Depression Scale was 35/80 (Evaluation of the level of depression: 20–44 normal range, 45–59 mildly depressed, 60–69 moderately depressed, 70 and above severely depressed), and the Kessler 6 Psychological Distress Scale for mood anxiety disorder questionnaire was 2/24. (Measure of psychological distress: A minimum score of 0 and a maximum score of 24. Low scores indicate low levels of psychological distress and high scores indicate high levels of psychological distress). Hematologic laboratory tests revealed mild renal impairment with a blood urea nitrogen level of 13.0 mg/dL, creatinine level of 1.26 mg/dL, and estimated glomerular filtration rate of 47.7 mL/min/1.73 m^2^, but no other abnormalities were observed in liver function or coagulability.

Considering his occupation as a truck driver, we decided not to prescribe any medications that could cause drowsiness, and prescribed duloxetine 20 mg/day (at bedtime) and acetaminophen 1200 mg/day. At the time of his visit 7 days after the start of oral therapy, his NRS was about 3 to 4/10 without much change, and the pain during driving continued. Therefore, we concluded that interventional treatment was necessary, and attempted ulnar and median nerve blocks through the left forearm approach. The needle was advanced under ultrasound guidance, and a mixture of 0.75% ropivacaine and 0.5% lidocaine (5 mL each) was administered to the ulnar and median nerves. For oral medication, the same amount of acetaminophen was continued, and the dose of duloxetine was increased to 40 mg (at bedtime). At the time of his visit 7 days after the nerve block was performed, his pain had improved to NRS 1 to 2/10, and the pain while driving had almost disappeared. The same nerve block was repeated, and 4 units of extract from inflammatory rabbit skin inoculated by vaccinia virus (Neurotropin®) was added to the oral medication. (This drug has been widely used as an analgesic in Japan for the treatment of chronic pain and neuropathic pain. And its use be authorized^2^). After 21 days from the first visit to our hospital, the pain had improved to NRS 1/10, and the medication alone was continued without any additional nerve blocks. The subsequent 21 days passed with his pain at NRS 0 to 1/10, and he was continued on the same dose of oral medication. He had virtually no pain while driving and no problems with his work. However, this was only a short‐term follow‐up, and there is the possibility that the pain will recur in the future.

## 3 DISCUSSION

Phantom limb pain and stump pain, which are considered to occur in 50%–80% of limb amputees, are intractable conditions.^1^ A study by Kooijman et al. evaluating the association between phantom limb pain and stump pain in 124 subjects with upper extremity amputations found a significant association between these two types of pain.^3^ Phantom limb pain is thought to be caused by several factors, including abnormal impulses from neuromas caused by peripheral nerve damage, and neuronal hyperexcitability at the level of the spinal cord.^4^ Phantom limb pain includes pain associated with motor or deep sensation, and pain associated with cutaneous superficial sensation.^5^ Phantom limb pain is different from stump pain, which is caused by pain local to the stump, but these amputation‐associated pain symptoms are often difficult to distinguish in clinical practice. Our present patient was also considered to have a mixture of these two types of pain.

There are several treatments for these types of pain, including antidepressants, antiepileptic drugs, opioids, and other medications, as well as surgical treatments, such as nerve blocks, but no standard treatment has been developed to date.^6^ Therefore, it is necessary to select an appropriate treatment for each patient. In our case, the patient's pain could not be controlled using medication alone, so nerve blocks were additionally performed. Mirror therapy is said to be effective for phantom limb pain.^7^ Under this therapy, a patient is allowed to feel the imaginary movement of the removed body part behaving as normal body movement through a mirror. The mirror image of the normal body part helps reorganize and integrate the mismatch between proprioception and visual feedback of the removed body. Thus, enhancing the treatment effect for phantom limb pain. But mirror therapy was not performed in our patient. There are also reports that transcutaneous electrical nerve stimulation (TENS) is effective for both phantom pain and stump pain.^8^ However, there has been no systematic review of evidence for the therapeutic effects of TENS. Therefore, the efficacy of TENS for phantom and stump pain remains unclear at present.^9^ In the present patient, we also investigated the possibility of brachial plexus block as an interventional treatment. However, because the pain was localized to the annular finger, and because a brachial plexus block would cause residual nerve damage in the thumb to the index finger, which could affect his job performance, we chose peripheral ulnar nerve block and median nerve block. In fact, the pain was markedly improved by the nerve blocks, and hence the pain was thought to be caused by pain in the ulnar and median nerve regions. Following the nerve blocks, his pain was controlled with oral medication alone, indicating that peripheral nerve blocks are effective for pain associated with cutaneous sensations, such as electrical shock pain and allodynia. There are few reports to date of the improvement of phantom limb pain or stump pain in the hand using peripheral nerve blocks, and our present case suggests that peripheral nerve block is a useful therapy for mixed phantom limb and stump pain.

Previous reports in the literature have demonstrated that intravenous lidocaine is effective for stump pain,^10^ and pulsed radiofrequency is effective as an interventional treatment.^11^, ^12^, ^13^ If the patient's pain relapses in the future, these therapies are an option. In addition, it is important to treat refractory phantom limb pain and stump pain using a variety of approaches, including psychotherapy and cognitive behavioral therapy.^14^

## 4 CONCLUSION

We encountered a case of a patient in whom peripheral nerve blocks were useful for mixed phantom limb and stump pain. Our case demonstrates that it is necessary to select a customized treatment method according to each patient's background and/or symptoms, as there is no established treatment for this type of pain.

## AUTHOR CONTRIBUTIONS

**Shunya Sekiguchi:** Conceptualization; writing – original draft. **Yusuke Ishida:** Conceptualization; writing – original draft; writing – review and editing. **Yosuke Fujita:** Writing – review and editing. **Mikiko Tomino:** Writing – review and editing. **Kiyoshige Ohseto:** Conceptualization; writing – review and editing.

## FUNDING INFORMATION

None.

## CONFLICT OF INTEREST STATEMENT

The authors declare that they have no competing interests associated with this manuscript.

## CONSENT

Written informed consent was obtained from the patient for publication of this case report and the accompanying images.

## Data Availability

None.

## References

[1] Aternali A , Katz J . Recent advances in understanding and managing phantom limb pain. F1000Res. 2019;8: F1000 Faculty Rev‐1167. doi:10.12688/f1000research.19355.1 PMC6652103 31354940

[2] Sumitani M , Sakai T , Matsuda Y , et al. Executive summary of the clinical guidelines of pharmacotherapy for neuropathic pain: second edition by the Japanese Society of Pain Clinicians. J Anesth. 2018;32(3):463‐478. doi:10.1007/s00540-018-2501-0 29737410 PMC5973958

[3] Kooijman CM , Dijkstra PU , Geertzen JH , Elzinga A , Van der Schans CP . Phantom pain and phantom sensations in upper limb amputees: an epidemiological study. Pain. 2000;87(1):33‐41. doi:10.1016/s0304-3959(00)00264-5 10863043

[4] Flor H , Nikolajsen L , Staehelin JT . Phantom limb pain: a case of maladaptive CNS plasticity? Nat Rev Neurosci. 2006;7(11):873‐881. doi:10.1038/nrn1991 17053811

[5] Karkin‐Tais A , Muftic M , Suljevic S , Hadziahmetovic N , Miladinovic K , Alajbegovic A . 13‐Year study of pain in phantom limbs of amputees–victims of war in Sarajevo. Eur J Pain. 1992–2005;10(S1), S98b‐S98. doi:10.1016/S1090-3801(06)60370-4

[6] Boomgaardt J , Dastan K , Chan T , Shilling A , Abd‐Elsayed A , Kohan L . An algorithm approach to phantom limb pain. J Pain Res. 2022;26(15):3349‐3367. doi:10.2147/JPR.S355278 PMC9618240 36320223

[7] Erlenwein J , Diers M , Ernst J , Schulz F , Petzke F . Clinical updates on phantom limb pain. Pain Rep. 2021;6(1):e888. doi:10.1097/PR9.0000000000000888 33490849 PMC7813551

[8] Mulvey MR , Radford HE , Fawkner HJ , Hirst L , Neumann V , Johnson MI . Transcutaneous electrical nerve stimulation for phantom pain and stump pain in adult amputees. Pain Pract. 2013;13(4):289‐296. doi:10.1111/j.1533-2500.2012.00593.x 22935086

[9] Johnson MI , Mulvey MR , Bagnall AM . Transcutaneous electrical nerve stimulation (TENS) for phantom pain and stump pain following amputation in adults. Cochrane Database Syst Rev. 2015;18(8):CD007264. doi:10.1002/14651858.CD007264.pub3 PMC7209768 26284511

[10] Wu CL , Tella P , Staats PS , et al. Analgesic effects of intravenous lidocaine and morphine on postamputation pain: a randomized double‐blind, active placebo‐controlled, crossover trial. Anesthesiology. 2002;96(4):841‐848. doi:10.1097/00000542-200204000-00010 11964590

[11] Hsu E , Cohen SP . Postamputation pain: epidemiology, mechanisms, and treatment. J Pain Res. 2013;6:121‐136. doi:10.2147/JPR.S32299 23426608 PMC3576040

[12] Zheng B , Song L , Liu H . Pulsed radiofrequency of brachial plexus under ultrasound guidance for refractory stump pain: a case report. J Pain Res. 2017;10:2601‐2604. doi:10.2147/JPR.S148479 29158692 PMC5683784

[13] Imani F , Gharaei H , Rezvani M . Pulsed radiofrequency of lumbar dorsal root ganglion for chronic postamputation phantom pain. Anesth Pain Med. 2012;1(3):194‐197. doi:10.5812/kowsar.22287523.3768 24904793 PMC4018701

[14] Modest JM , Raducha JE , Testa EJ , Eberson CP . Management of post‐amputation pain. R I Med J (2013). 2020;103(4):19‐22. 32357588

